# Characteristics of Psychiatric Visits to the Emergency Department of Rasoul-e-Akram Hospital, Tehran, Iran

**Published:** 2012

**Authors:** Atefeh Ghanbari Jolfaei, Mehdi Nasr Isfahani, Fatemeh Shoyookhi

**Affiliations:** 1 Tehran University of medical sciences, Tehran, Iran.

**Keywords:** Emergency, Indication, Psychiatric Visit

## Abstract

**Background: **More psychiatric visits, especially non-emergency ones, to emergency departments (EDs) of general hospitals have been observed in recent years. The aim of this study was to determine the characteristics of psychiatric visits to the ED of Rasoul-e-Akram Hospital, Tehran, Iran.

**Methods: **In this cross-sectional study, during a two-month period, all psychiatric presentations and consultations to the ED of the studied hospital were included. The required data were gathered by psychiatry chief residents and were documented in pre-designed checklists.

**Results: **About 0.01% of all patients presenting to the ED needed the psychiatric visits. Men consisted 50% of the total patients with mean (±SD) age of 36.41 (±14.7) years. About 51% of them had the indication of the emergency psychiatric visit while 47% had the indication of hospitalization in the psychiatric ward. Non-emergency visits were not related to demographic characteristic, previous psychiatric disorders, substance abuse and physical diseases

**Conclusions: **Non-emergency visits take a high percentage of psychiatric visits in ED and regarding limited sources for psychiatric emergencies and Long visiting time, this percentage can hinder the process of giving services to real emergency psychiatric patients.

## Introduction

The number of psychiatric patients presenting to EDs has increased in different countries in recentyears. Similarly, there has been a 38% increase in psychiatric visits toEDs in the US from 1992 to 2001(1). In addition, a study from NationalAmerican College of Emergency Physicians demonstrated a 61.3% increase in patientspresenting to psychiatric emergencies EDs in a course of 6 to 12 months (2). Besides in other countries, psychiatric visits to EDs have increased due to an increase in the number of psychiatric units of general hospitals (3-5).

Regarding this increase, many studies have been carried out about demographic characteristics, symptoms and disorders, the reasons of referrals and admission indications ofmental health patients in EDs in many countries (6-9).

As far as we know, there are few similar studies in our country about the percentage and characteristics of psychiatric visitsto the EDs of general hospitals. In this study, the authors intended to investigate somecharacteristics of psychiatric visits to the ED of Rasoul-e-Akram hospital. This general hospital has affiliation to Tehran University of Medical Sciences, Tehran, Iran. This center has no selective admission policy and admits patients from all over the country. 

## Materials and Methods

In this cross-sectional study, all emergency psychiatric visits to the studied hospital in a two-month period were studied. The data was collected by psychiatric chief residents, who had passed courses in diagnosing and treating emergency psychiatric problems, and recorded the required data insidepre-designed checklists made by the researchers. The data consisted of demographic characteristics, the time of request ofvisit, length of each visit, past history of psychiatric disorders, medical disease, substance abuse, emergency visit indication, need of hospitalization and its reason, need of revisiting and its reason and the process of discharging the patient. The information was gathered by interviewing the patient and their relatives and reviewing theirfiles. The mean duration of information gathering time was 45 minutes. The emergencyvisit indication was determined based on clinical judgments of psychiatric residentsand indications such as the danger of self-harm and harm to others, crisis encounter, symptoms of substance intoxication or withdrawalor treatment side-effects requiring immediate intervention.

The need for hospitalization was also determined based on indications such as self-harm or harm to others, not responding to outpatient treatments, not having enough support or existence of environmental psychological stresses, especial treatment aims and judicial orders.

To analyze data,using the SPSS software for Windows (Version 15), and descriptive indices were used to express data and the student t-test and logistic regression were usedto interpret data.

## Results

In a course of 2 months, 102 psychiatric visits were done in the ED of Rassoul-e-Akram hospital which was 0.01% of all 10124visits to the ED of this hospital.There were 51 men (50%) and 51 women (50%). The averageage of patients was 36.41±14.7years (range, 14-73). Other demographic characteristics of patients such as marital status, occupation and educational level are shown in table 1.

**Table1:** Demographic features of mental health-related ED visits

**Variables**	**Frequency (%)**
**Marital status**	
Married	52(51)
Single	45(44)
Divorced	3(3)
Widow	2(2)
**Occupational status**	
Employed	42(41.2)
Unemployed	60(85.8)
**Educational status**	
Illiterate	1(1)
Primary education	4(3.9)
Secondary education	25(24.5)
High school before diploma	50(49)
High school Diploma and college	22(21.6)

Mean time of calling the resident and starting the visit was 14.6 ±5.9 minutes (range, 10-40 minutes). Meanlength of visiting time was 41.68 ±22.36 minutes (range, 5-120).

Forty-six patients (45.1%) had previously diagnosed by psychiatric disorders, 31 cases (31.4%) had pervious or current substance abuse and 30 cases (29.4%) had medical diseases.

Fifty-two patients (51%) had indication of emergency psychiatric visit and the remainder (50 patients, 49%) either came to the ED for non-emergency reasons or emergency physicians requested psychiatric consultationfor non-emergency conditions. The frequency distribution ofemergency visit indication based on other variables such as age, gender, educational level, and so on is shown in table 2.

Twenty-eight patients (27.45%) were asked to present for a second visit, 9 patients (32%) for further diagnostic investigations,8 cases (28.6%) with suspicion of having organic problems, 8 patients (28.6%) for not having good conditions for the interview due to the low consciousness or emergency medical problems, and 3 patients (10.8%) for other reasons.

**Table 2:** The frequency distribution of emergency visit indication based on other variables

**Variables**				
	**Yes**	**No**	**P value**	**df**
**Gender (%)**				1
Female	25(48% )	26 (52%)	0.295	
Male	27 (52%)	24 (48%)		
**Age: mean (SD)**	33.8±12.9	±12.2 32.2	0.166	6
**Educational status **				4
Illiterate	0(0)	1(2%)		
primary education	2(3.8%)	2(%4(		
secondary education	15(28.8%)	10(20%)	0.706	
high school	24(46.2%)	26(52%)		
diploma and college	11(21.2%)	11(22%)		
**Occupational status **				1
Employed	21(40.4%)	22(44%)	0.22	
Unemployed	32(59.6%)	28(%56)		
**Marital status **				3
Married	25(%48.1(	27(%54(		
Single	23(%44.3)	22(%44)	0.528	
Divorced	2(%3.8)	1%)2)		
Widow	2(%3.8)			
**Previous psychiatric disorders**	30(%57.7)	16(%32(	0.56	1
**Previous or current substance abuse**	18(%34.6(	13(%26)	0.659	1
**Comorbid medical diseases**	14(26.9%)	16(32%)	0.2	1

Forty-eight patients (47%) had indication to be hospitalized in psychiatric ward of the hospital (Table 3).

**Table3:** Indication of hospitalization and the reason of not hospitalizing

**Variables **	**Frequency (%)**
**Indication of hospitalization **	
The risk of self-harm	29 (60.42)
The risk of harm to others	25 (52.08)
Not responding to outpatient treatments	15 (31.25)
Special treatment aims	07(14.58)
Drug side effects (ex. NMS)	07 (14.58)
existence of environmental psychological stresses	03 (6.25)
Not having enough support	01 (2.08)
**Hospitalization**	
Yes	32 (66.7)
No	16 (33.3)
**The reason of not hospitalizing**	
Not having enough beds	12 (75)
Not having facilities	03(18.75)
Family didn’t consent to hospitalization	01( 6.25)

The need to be hospitalized had no significant statistical relationship with age (p=0.024, df =6), level of education (p=0.866, df=4), marital status (p=0.2, df=3), gender (p=0.832, df=1), and the current substance abuse (p=0.426, df=1). However, need for hospitalization showed significant relationship with occupation (p<0.01, df=1), medical disease (p<0.01, df=1), and pervious psychiatric disorders (p<0.01, df=1), in a way that more hospitalization indications were observed in unemployed peopleand patients with medicaldiseases or past history of psychiatric disorderscompared to those who did not met these criteria (Table4).

**Table 4:** The frequency distribution of hospitalization indication based on other variables

**variables **	**Indication of hospitalization**			
	**Yes**	**No**	**P value**	**df**
**Gender (%)**				1
Male	25(52.08)	26(48.15)	0.832	
Female	23(48.92)	28(51.85)		
**Age: mean (SD)**	31.5(12.2)	39.2 (14.8)	0.024	6
**Educational status**				4
Illiterate	0) 0(	01.85) 1(		
primary education	06.25) 3 (	01.85) 1 (		
secondary education	25)12(	13(24.07)	0.866	
high school	50)24(	26(48.15)		
diploma and college	18.75) 9(	13(24.07)		
**Occupational status**				1
Employed	29.17)14 (	28(51.85)(	0.01	
Unemployed	70.83)34 (	26(48.15))		
**Marital status **				3
Married	23(47.92)	29(53.70)		
Single	22(45.83)	23(42.6)	0.2	
Divorced	02(4.17)	01(1.85)		
Widow	01(2.08)	01(1.85)		
**Previous psychiatric disorders**	30(62.5)	16(29.63)	0.002	1
**Previous or current substance abuse**	17(35.42)	14(25.92)	0.426	1

## Discussion

The present study showed that of all emergencyvisits, 0.01% was due to psychiatric reasonswhich is much less than reported figures fromother countrieswhichis between 2 and 2.5%. The reason for this considerable difference is not related to number of psychiatric visits because in other countries the average visits was between 60-90 people per month in comparison with the 50 people in our study. However the real reason maybe is the great number of visitors to theED of the Rasoul-e-Akram Hospital as a major hospital in Tehran (1, 4, 8).In this study, only 51% of patients had the indication of emergency psychiatric visit.Itis very common to see non-urgent use of EDs in many parts of the world and all medical fieldsandit is reported that such visits take 85-95% of visits.Surprisingly, outpatient visits had a 50% increase from 1955 to 1970, whereas emergency visits increased by312% in the same period in the US(10).Although 52% is more heartwarming than 85-95 % for non-urgent medical visits in EDs in other countries, regardinglimited sources for psychiatric emergencies, long visiting times(which was in average 42 minutes in this study) and the need to have special facilities for such visits, this percentage can still hinder the process of giving services to real emergency psychiatric patients (11).

In this study, no demographic characteristicswere related to non-emergency visits to the ED. Results of most studies about the gender of non-emergency visits to emergency wards are different. Some, like the present study, showed no difference between genders (12, 13) and some have suggested that it is more common in men (14) or in women(15).Unlike the probability of having more visits from the older patients, in this and most studies, the age range of patients was between 20 and 40 years of age which is in accordance with the age range of all visitors to the EDs(12, 13).

In some studies it is reported that havinglow social support, living alone and being single are related to non-urgent use of EDs(16, 17). However, in this study, marital status was not related to this issue which is probably because of the fact that inour country the immediate family consisting father, mother and siblings is still a very important provider of support, and being single does not necessarily show isolation and lack of social support . It is clear that carrying out studies with more samples with the aim of analyzing social support with special questionnaires can clarify the relation between social support and non non-urgent use of EDs. In this study, job status as a mark of economic statuswas not in any relation with non-emergency psychiatric visits in the ED. Although some studies have shown economic status to berelated to non-urgent use of EDs, race influencesthis relation. As was seen, economic status in whites has a reverse relation with non-emergency visits in ED, but for blackseconomic conditions are not in any relation to non-emergency visits(12, 18).Onthe onehand,poverty and being covered by insurance are the benefits of emergency service especially for unemployed people with poor economic status. On the other hand, its being 24 hours has made it available for the people who work and cannotgo to outpatient centers during the day. 

Some of these visits may be requested by emergency physicians for medical patients, with non-urgent psychiatric signs and symptomsandeven 28.6% of needs to revisits were due to the unsuitable level ofconsciousness or the existence of medical emergencies. This emphasizes the impotence of increasing coordination and cooperation between emergency physicians and psychiatriststo reduce time and expenses. Furthermore, medical professionals and patients may differ in what they assume as a medical emergency is (19).This also may be true for emergency psychiatric situations and having more studies about public attitude toemergency psychiatric problem could be useful.

In this study, 47% of patients needed to be hospitalized which is very close to western countries(20) and differs from 77.9% in Khalili and Yasami’s study(21)which is probably because of not having as many samples as that study or maybe it is because of the sooner time of treatment in our samples due to better socioeconomic conditions based on their geographical location, because in that study this differs between two hospitals too and 68.4% of patients in Taleg\any and 80.1% of patientsin Imam Hosein hospital needed hospitalization, although differences between the two hospitals were not statically meaningful. Furthermore, there have been better outpatient psychiatric services and less stigma of visiting psychiatrist in recent years.

In this study, 75% of patients were not hospitalized due to insufficient beds and 18.7% for not havingessential facilities like isolation room for aggressive patients which is high percentage like the study of Khalili and Yesami(21)and necessitates the need to make more psychiatric wards and preventive services.

Like the study carried out by Khalili and Yasmi(21)and smith et al.(6), educational level, marital status, ageand gender were not related to the need to be hospitalized in this study,however, job status, medical disease and pervious psychiatric disorders were related, butunlike this study, it was shown that there is a direct relation between substance abuse and the need to be hospitalized in the mentioned studies. This is probably because of the fact that weincluded both current and previous substanceabusers in our study or it could be for the fact that we had fewer samples in comparison with those mentioned studies. 

The limitations of this study must be mentioned. First we did not evaluate some important variables like economic status and support system. Second, our sample was not large enough. Future studies should address these issues in larger sample sizes.

## Author’s Contributions

AGhJ and MNI conceived and designed the evaluation, and interpreted the clinical data. AGhJ performed parts of the statistical analysis and drafted the manuscript. FSh collected the clinical data and performed parts of the statistical analysis. All authors read and approved the final manuscript.

## Notes

**Declaration of interest:** None.
